# Supplementary material for: Multi-scale simulations of MUT-16 scaffold protein phase separation and client recognition
Source: Biophys J. 2025 Aug 7;124(22):3987–4004. doi: 10.1016/j.bpj.2025.08.001 (PMC12709416; doi:10.1016/j.bpj.2025.08.001)
Supplement: Document S1. Figures S1–S10 and Table S1 [file mmc1.pdf]

Biophysical Journal, Volume *124*

Supplemental information

Multi-scale simulations of MUT-16 scaffold protein phase separation and client recognition

Kumar Gaurav, Virginia Busetto, Diego Javier Páez-Moscoso, Arya Changiarath, Sonya M. Hanson, Sebastian Falk, René F. Ketting, and Lukas S. Stelzl

SUPPLEMENTARY MATERIAL

Supplementary Text

Since interactions in CALVADOS2 models are governed by residue specific λ-values (and electrostatics), which quantify the hydropathy driven stickiness of each amino acid, we sought to evaluate whether these values can recapitulate the interaction patterns observed in our simulation derived contact maps. We performed a correlation analysis between the simulation-derived contact map (Fig. S5A) and a lambda-based interaction profile (Fig. S5B) averaged per residue, yielding a modest Pearson correlation of r = 0.44 (Fig. S5C) at the residue-pair level. specific residue pairs such as Arg698 and Arg699 with Tyr and Glu appear as outliers in the correlation plot, deviating significantly from the overall trend. Aggregating to the amino acid level the contact maps ((Fig. S5D,E) revealed an improved the correlation to r = 0.76 (Fig. S5F), indicating that lambda values reasonably capture overall interaction tendencies. However, specific interactions involving charged and flexible amino-acids (e.g., Arg-Asp, Arg-Glu, Lys-Asp, Lys-Glu, Gly-Gly, Gly-Tyr, Gly-Phe, Gly-Leu, Gly-Ser, and, Gly-Cys) were poorly represented, underscoring the limitations of lambda-derived propensities in capturing detailed contact behavior.

Supplementary Table

Model	Force-field	Number of protein chains	Box dimension (nm)	Number of particles	Time
Atomistic	Amber99sb-star-ildn-q	100 (MUT-16 M8BR)+10 (MUT-8)	15×15×30	884932	1 µs
Atomistic (Folding@home)	Amber99sb-star-ildn-q	100 (MUT-16 M8BR)+10 (MUT-8)	15×15×30	884932	350 µs
Near-atomic coarse-grained	Martini3 (@λ = 1,1.01,1.03,1.05)	100 (MUT-16 M8BR+FFR)	40×40×40	657674	10 µs
Near-atomic coarse-grained	Martini3 (@λ = 1,1.01,1.03,1.05)	180 (MUT-16 FFR)	40×40×40	659058	10 µs
Near-atomic coarse-grained	Martini3 (@λ = 1,1.01,1.03,1.05)	220 (MUT-16 M8BR)	40×40×40	658434	10 µs
Near-atomic coarse-grained	Martini3	100 (MUT-16 M8BR+FFR)	60×60×60	1675095	20 µs
Near-atomic coarse-grained	Martini3	100 (MUT-16 M8BR+FFR)+20 (MUT-8)	60×60×60	1676715	20 µs
Near-atomic coarse-grained	Martini3	100 (MUT-16 M8BR)+10 (MUT-8)	15×15×60	138557	20 µs
Near-atomic coarse-grained	Martini3 (@λ = 1.03)	100 (MUT-16 M8BR)+10 (MUT-8)	15×15×60	138557	20 µs
Residue-level coarse-grained	CALVADOS2	100 (MUT-16 M8BR +FFR)	20×20×280	31200	10 µs
Residue-level coarse-grained	CALVADOS2	220 (MUT-16 M8BR)	20×20×280	30800	10 µs
Residue-level coarse-grained	CALVADOS2	180 (MUT-16 FFR)	20×20×280	30960	10 µs
Residue-level coarse-grained	CALVADOS2 (@T = $260K, 265K, 270K, ..., 285K$)	100 (MUT-16 M8BR +FFR)	20×20×150	31200	10 µs
Residue-level coarse-grained	CALVADOS2	100 (MUT-16 M8BR +FFR)+10 (MUT-8)	20×20×280	31710	10 µs

Table S1: Simulation characteristics. Listed here are the various models and force-fields used to perform the simulations. Furthermore, the box dimensions, number of particles, type and number of proteins, and simulation time are also listed.

Supplementary Figures

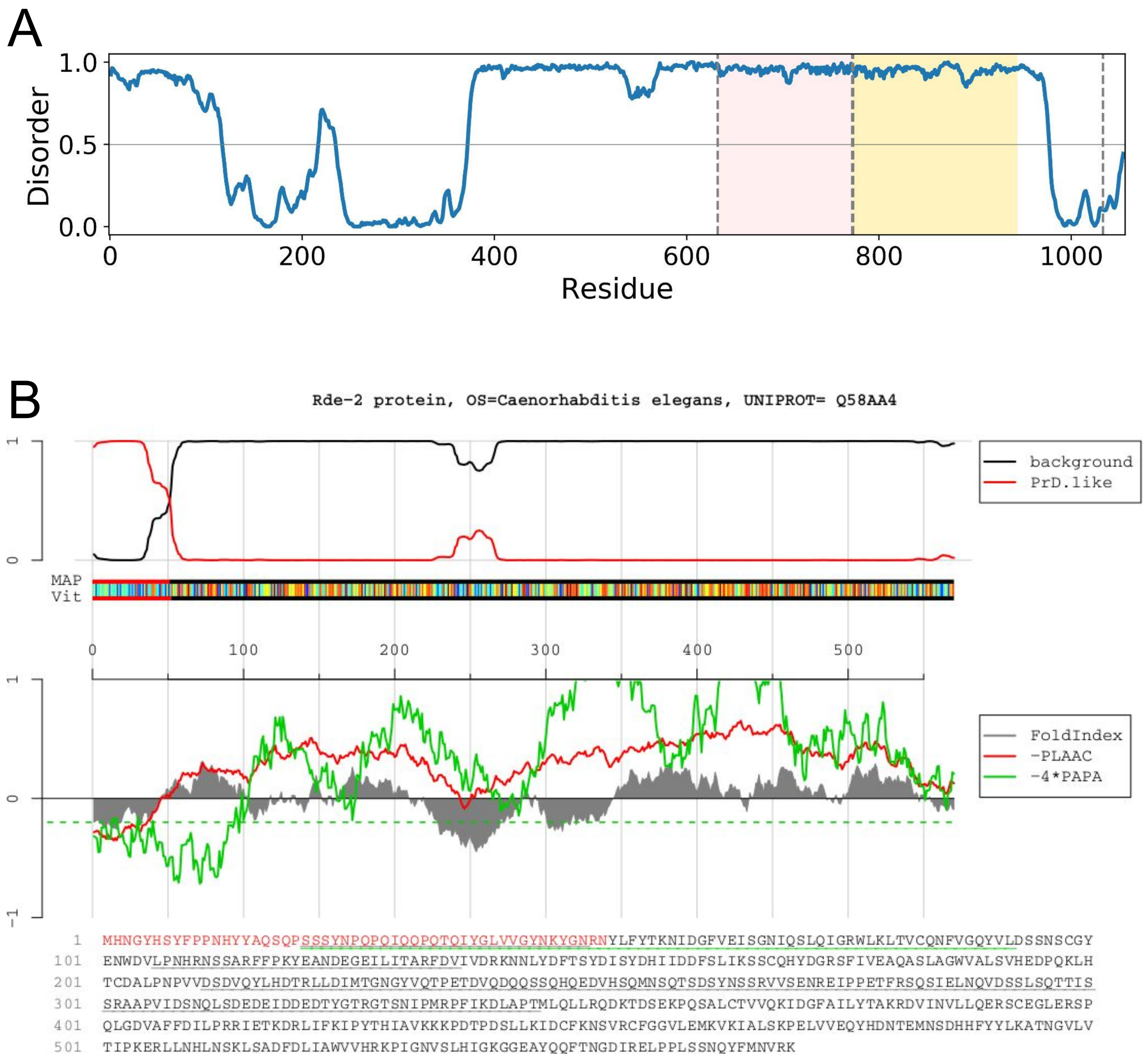

Figure S1: **A.** Disorder prediction for MUT-16, the M8BR (pink) and FFR (yellow) regions are highlighted. **B.** Prediction of the prion-like domain (PLD) in MUT-8 protein using PLAAC webserver(**?**)

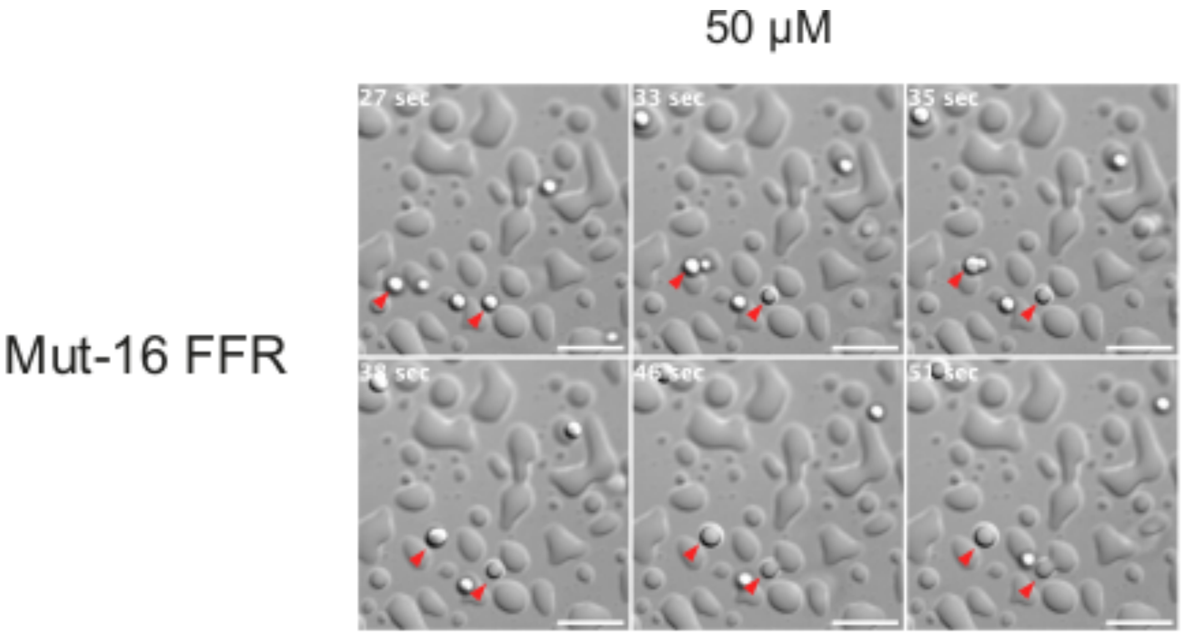

Figure S2: Time-lapse analysis demonstrates the fusion of phase-separated droplets formed by the addition of 50 μM MUT-16 FFR and a 1:100 ratio (w/w) of 3C protease to the MUT-16 fragment. Red arrowheads indicate droplets merging into larger ones and wetting the coverslip surface. Time refers to the imaging period. Scale bars: 10 μm.

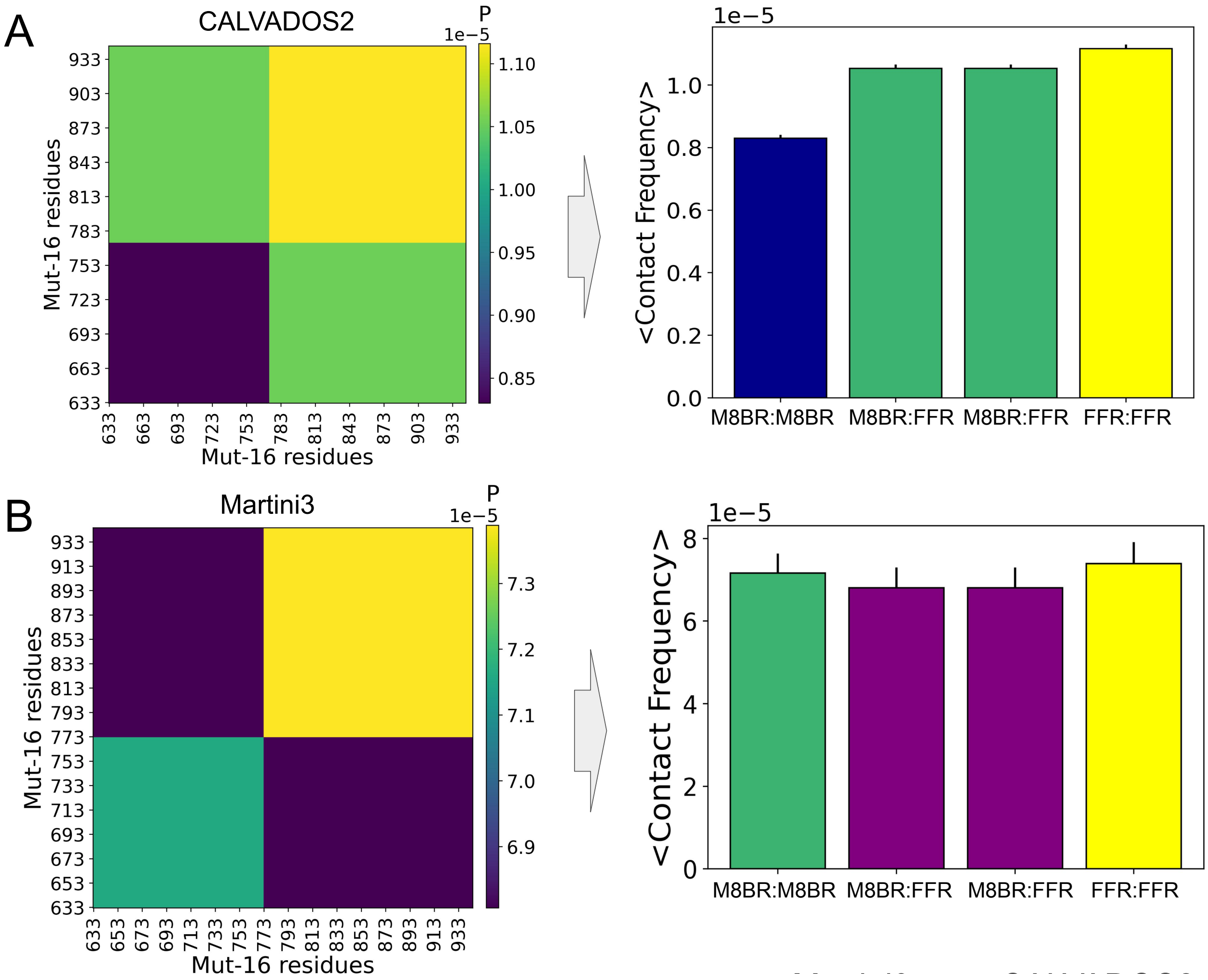

Figure S3: **A.** Region-wise contact frequency calculated for M8BR:M8BR (blue), M8BR:FFR (green) and FFR:FFR (yellow) interactions in residue-level coarse-grained simulation (CALVADOS2), Bar plot of average contact values from each region calculated from the CALVADOS2 simulations. **B.** Region-wise contact-map obtained for M8BR:M8BR (green), M8BR:FFR (purple) and FFR:FFR (yellow) interactions in near-atomic coarse-grained Martini3 simulations, Bar-plot of average contact frequency of interactions between different regions obtained through Martini3 simulations.

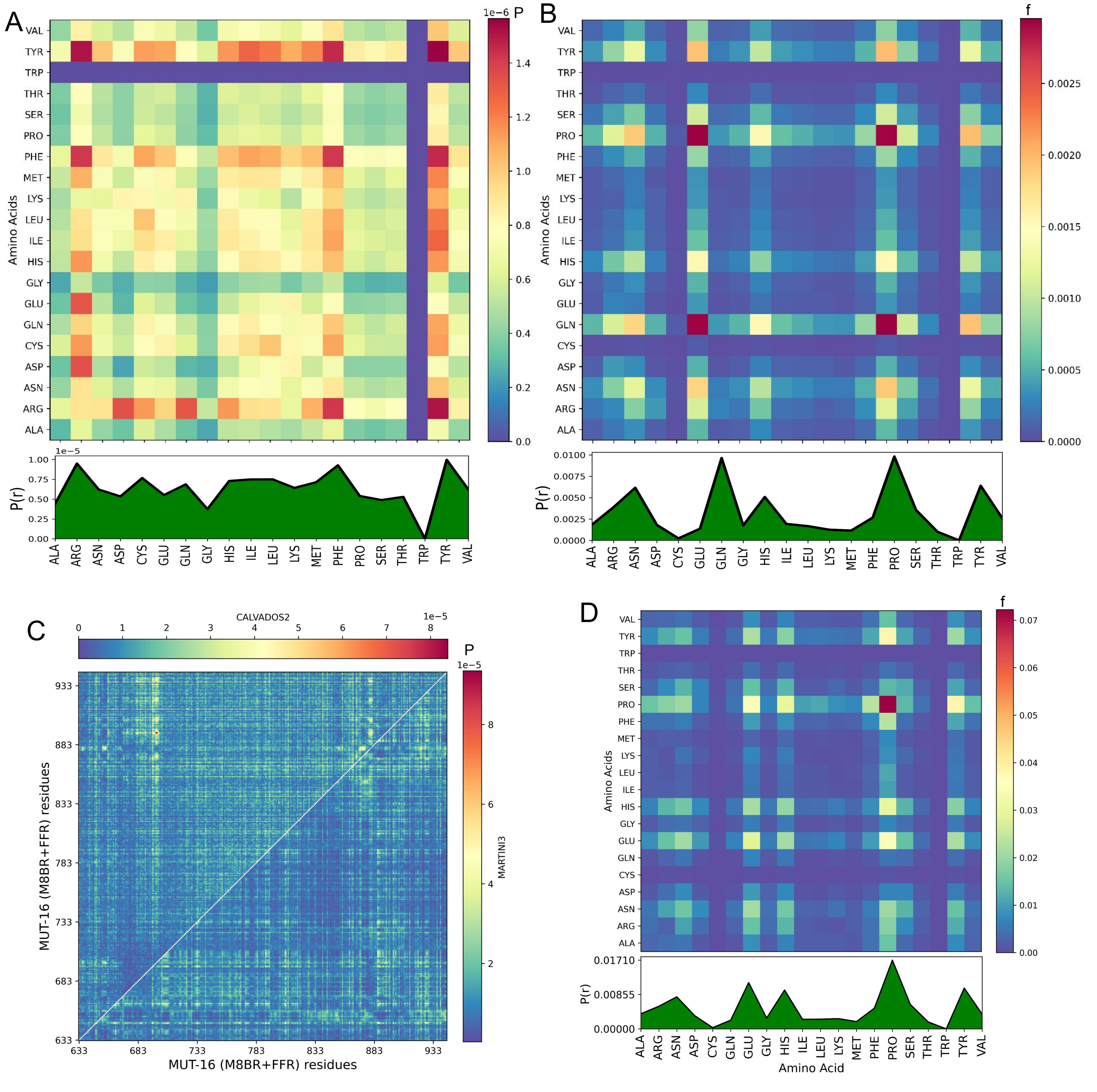

Figure S4: **A.** Amino acid contact map from CALVADOS2 simulations, normalized by the number of residue-residue pairs for each amino acid combination. **B.** Unnormalized amino acid contact map from CALVADOS2 simulations. **C.** Composite contact map showing CALVADOS2 data in the upper triangle and MARTINI3 data in the lower triangle, enabling direct comparison. **D.** Unnormalized amino acid contact map from MARTINI3 simulations.

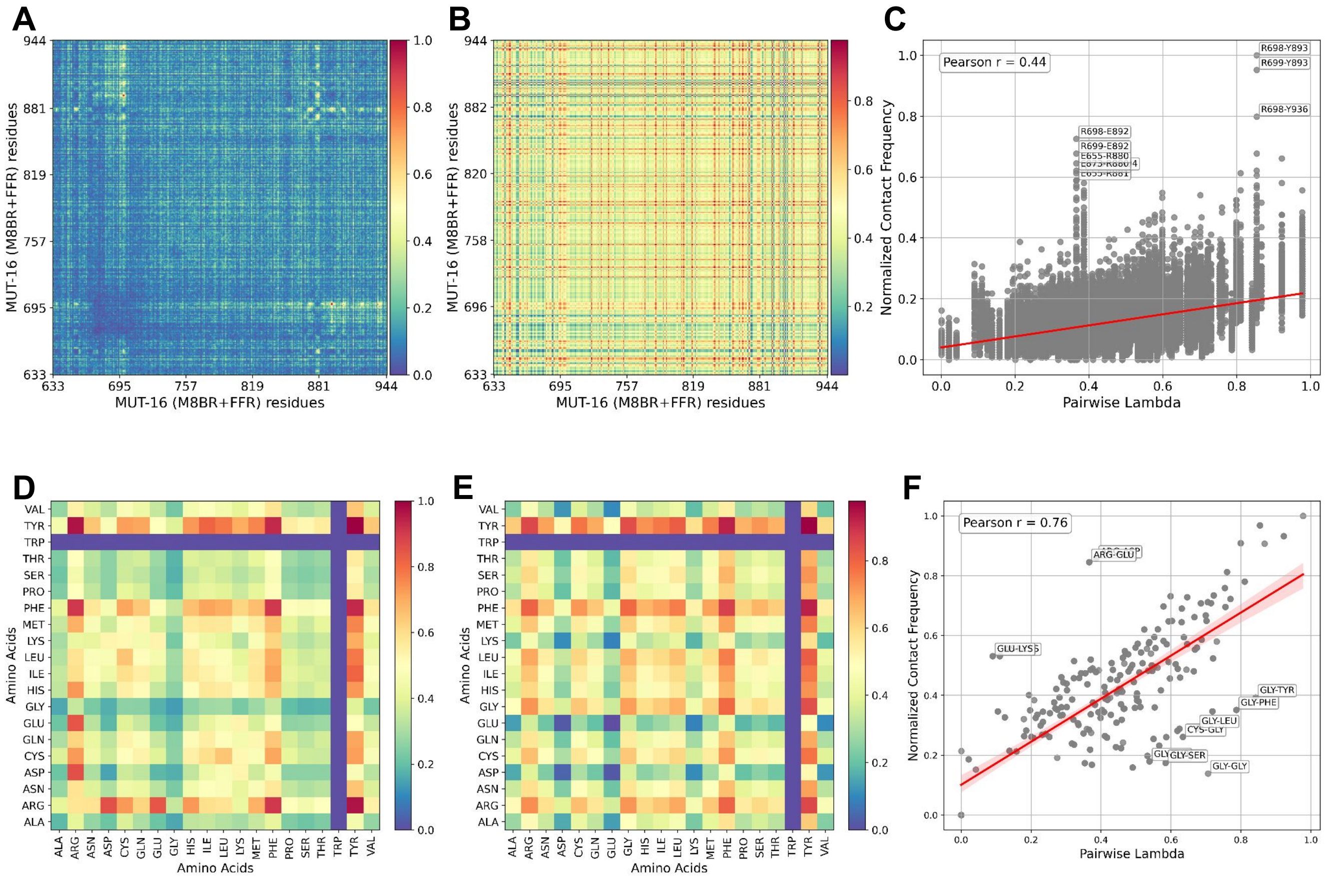

Figure S5: Correlation analysis between simulation-derived contact maps and lambda-based interaction profiles. **A.** Normalized contact map from simulations (min-max scaling). **B.** Interaction profile obtained by averaging lambda values for each amino acid. **C.** Correlation between **A.** and **B.**, yielding a Pearson coefficient of r = 0.44. **D.** Amino acid-level contact map. **E.** Lambda-based interaction profile at the amino acid level. **F.** Correlation between **D.** and **E.**, with a Pearson coefficient of r = 0.76.

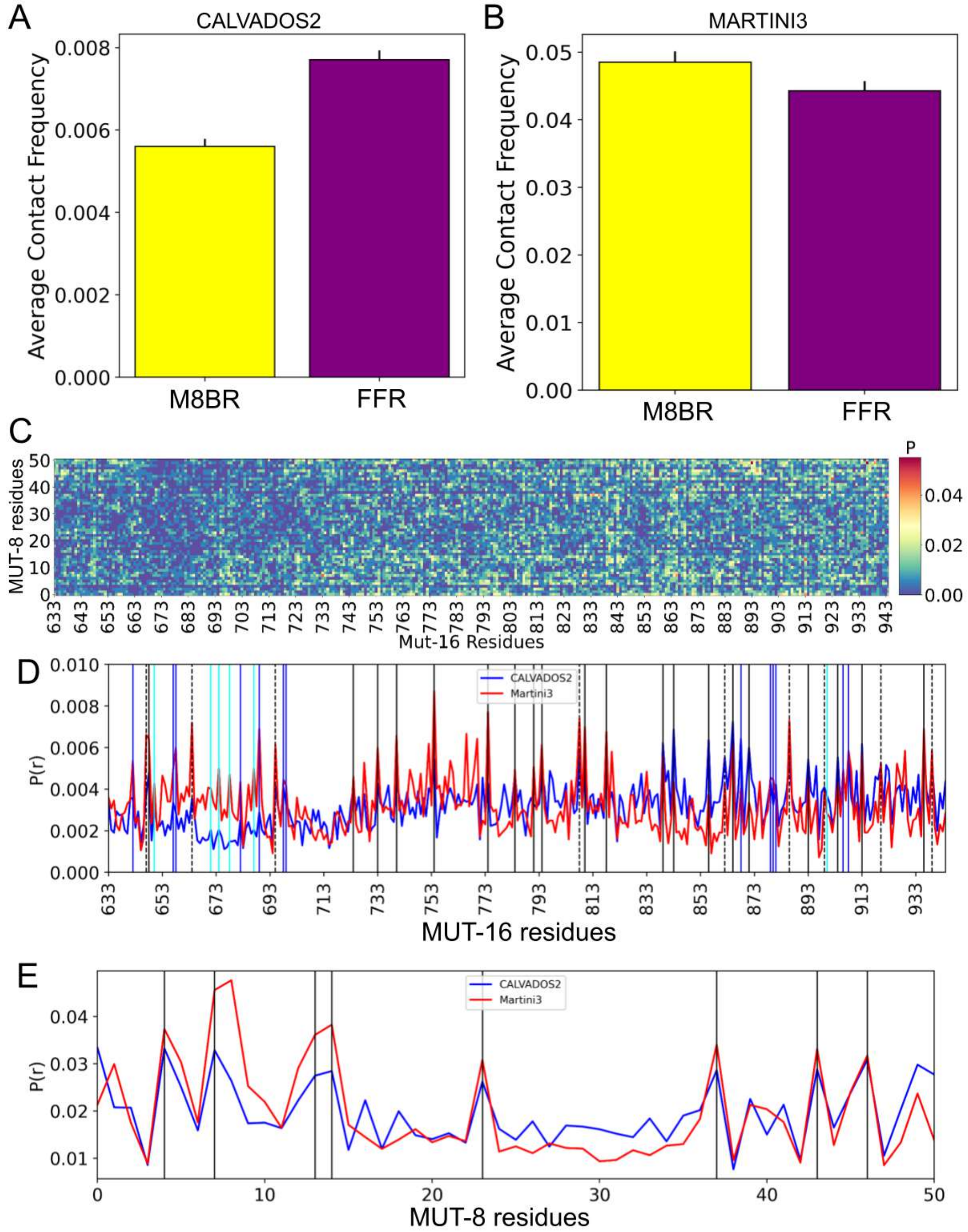

Figure S6: **A,B.** Average contact frequency of MUT-16 M8BR+FFR with MUT-8 N-terminal in CALVADOS2 and Martini3 simulations, respectively. **C.** 2-D contact map of interaction between MUT-8 and MUT-16 obtained from CALVADOS2 simulations. **D.** 1-D contact plot of MUT-16 (M8BR+FFR) obtained through Martini3 (red) and CALVADOS (blue), showing the relative frequencies of making contact per residue. Aromatic and positively charged amino acids like Tyr (black), Phe (black, dashed), Arg (blue), and Lys (cyan) are represented by the verticle lines. **E.** 1-D contact plot of MUT-8 N-terminal obtained through Martini3 (red) and CALVADOS2 (blue), showing the relative contact frequencies per residue.

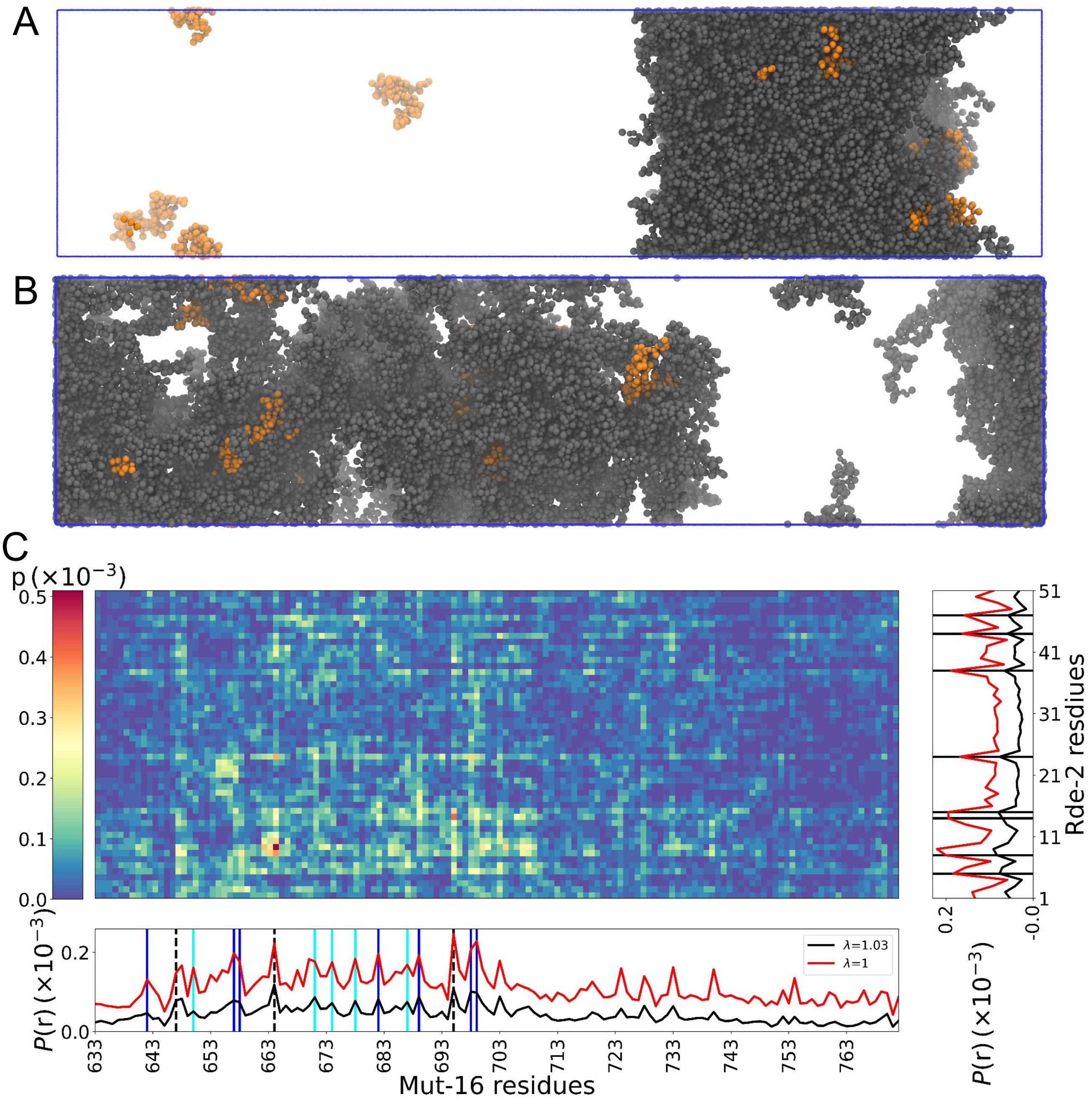

Figure S7: **A,B.**Final frame of the simulation for MUT-16 M8BR and MUT-8 N-terminal at λ = 1 and λ = 1.03. **C.** 2-D inter-molecular contact map of MUT-16 M8BR and MUT-8 at λ = 1.03. 1-D contact map at λ = 1.03 is compared to the simulation profile at λ = 1.

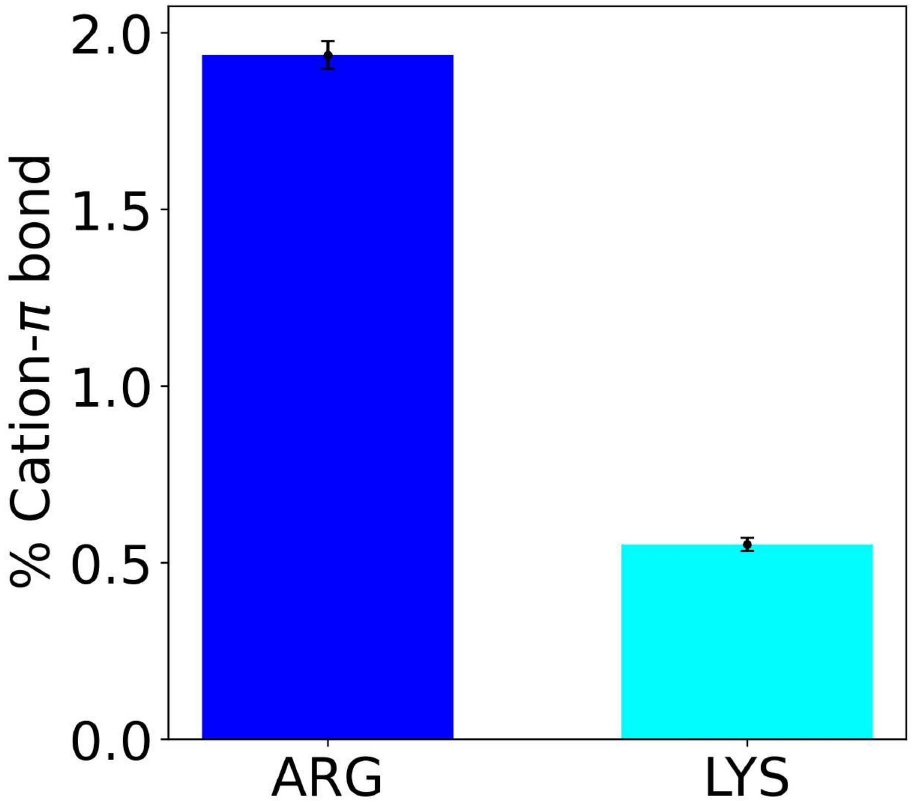

Figure S8: Percentage of cation-π interactions between Arg/Lys of MUT-16 M8BR and Tyr of MUT-8 N-terminal domain with the Martini3 simulation model. Percentage is relative to the total amount of possible pairwise Arg and Lys Tyr interactions

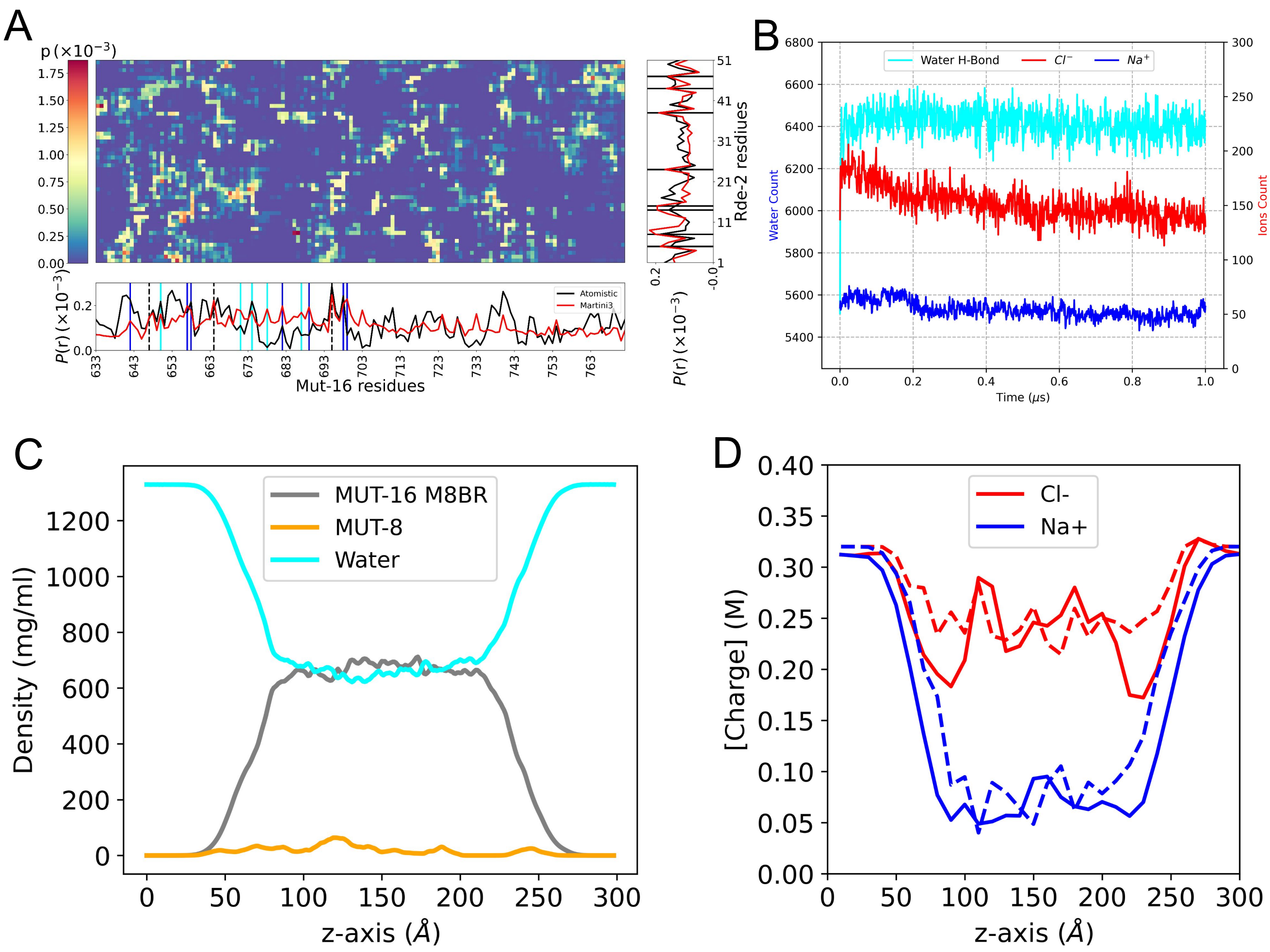

Figure S9: **A.** Contact map obtained from atomistic simulation of MUT-16 M8BR cluster and MUT-8 N-terminal PLD. 1-D contact map obtained by atomistic simulation and Martini3 simulations are compared. Residues like Tyr (black), Phe (dashed), Arg (blue), Lys (cyan) are indicated with vertical lines. **B.** Interactions of water and ions with MUT-16 M8BR during the 1 μs trajectory are shown. The cyan line represents the number of water molecules making hydrogen bonds with protein. The blue line represents the number of Na^+ ions bound to negatively charged residues. The red line represents the number of Cl^- ions in electrostatic interaction with the positively charged residues. For calculating the number of ions bound an interaction a cutoff of 5 Å between the charged atoms was used. **C.** Density profiles obtained from atomistic simulations. Components are shown in the legend. **D.** Density profile corresponding to Na^+ and Cl^- ions. Dashed lines represent the predicted values obtained from the concentration of cationic and anionic residues in both MUT-16 and MUT-8 proteins.

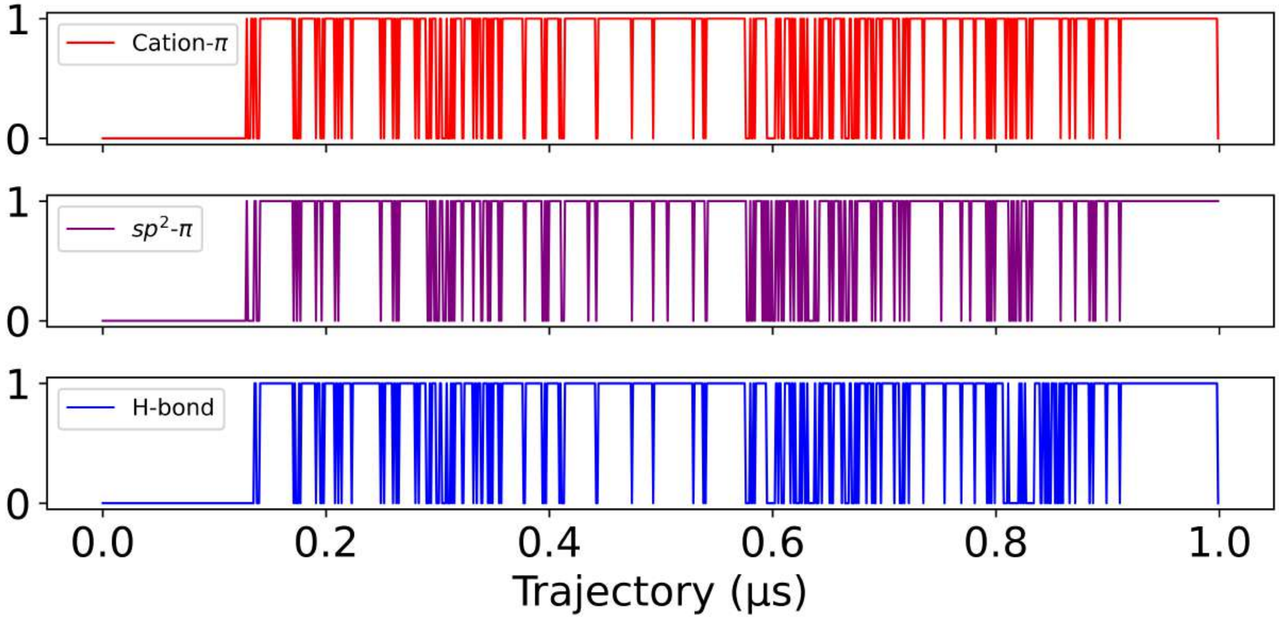

Figure S10: Different non-covalent interaction between a pair of Arg residues of MUT-16 M8BR and Tyr of MUT-8 N-terminal domain involving the cation-π, sp^2-π and H-bond (given the cation-π interaction has already formed).
